# Pharmacy Practice and Education in Austria

**DOI:** 10.3390/pharmacy6030055

**Published:** 2018-06-26

**Authors:** Thierry Langer, Helmut Spreitzer, Teresa Ditfurth, Gunar Stemer, Jeffrey Atkinson

**Affiliations:** 1 Department of Pharmaceutical Chemistry, Faculty of Life Sciences, University of Vienna, Althanstrasse 14, 2D 306, 1090 Vienna, Austria; thierry.langer@univie.ac.at (T.L.); Helmut.Spreitzer@univie.ac.at (H.S.); 2 Austrian Chamber of Pharmacists, Spitalgasse 3, 1090 Vienna, Austria; teresa.ditfurth@apothekerkammer.at; 3 Stellvertretender Apothekenleiter, Arzneimittelinformation und Klinische Pharmazie, AnstaltsapothekeAllgemeines Krankenhaus der Stadt Vienna–Medizinischer Universitätscampus, Währinger Gürtel 18-20, 1090 Vienna, Austria; gunar.stemer@akhwien.at; 4 Pharmacolor Consultants Nancy, 12 rue de Versigny, 54600 Villers, France

**Keywords:** pharmacy, education, practice, Austria, European Union

## Abstract

The PHARMINE (“Pharmacy Education in Europe”) project studied pharmacy practice and education in the European Union (EU) member states. The work was carried out using an electronic survey sent to chosen pharmacy representatives. The surveys of the individual member states are now being published as reference documents for students and staff interested in research on pharmacy education in the EU and in mobility. This paper presents the results of the PHARMINE survey on pharmacy practice and education in Austria. In the light of this, we examine the harmonisation of practice and education in Austria with EU norms.

## 1. Introduction

The PHARMINE (“Pharmacy Education in Europe”) consortium surveyed the state of pharmacy practice and education in the member states of the European Union (EU), including Austria, between 2008 and 2011, with an update in 2017. The methodology used in the PHARMINE study and the main results obtained have already been published [1]. In the first part of the study, PHARMINE gathered information on community practice, and on specialised hospital and industrial practice, as well as the necessary education and training. PHARMINE also dealt with other personnel working in pharmacies such as pharmacists’ assistants: their education, training and responsibilities.

PHARMINE went on to study the legal and administrative context of pharmacy practice and education. In the EU pharmacy practice and education fall under two jurisdictions: European and national. EU legislation is confederal in structure. Freedom of movement and of exercise of profession is the cornerstone. To ensure this there is a system of automatic recognition of professional qualifications for sectoral professions such as pharmacists. For regulated professions in some specific sectors—doctor, dentist, pharmacist, general care nurse, midwife, veterinary surgeon, architect and lawyer—recognition of a professional coming from an EU member state in other EU member states is regulated by the EU sectoral directives (for pharmacists, etc.) that are individual for each of the professions. For all other professions such restictions do not exist. To work in another EU member state, such professionals apply to the relevant authority of that country, providing proof of the qualifications obtained in their home state. Such procedures are regulated by directives issued by the European Commission of the EU. The latter are ordinances laying down the broad imperatives on the practice and education of the given profession [2]. An EU directive requires member states to achieve a particular result—in this case harmonisation of practice and education—without dictating the means of achieving that result. Thus directives leave the different member states with leeway as to the exact rules to be adopted. The result of this is that member states have systems that are more or less harmonised with the EU paradigm. 

In parallel to the above pan-national system, member states may introduce national legislation relating for example to specialised practice, and to ownership and management of pharmacies. 

Pharmacy education and training in Europe is also influenced by the Bologna agreement on the harmonization of European degree courses, and student and staff exchange [3]. The Bologna agreement was signed by the education ministers of the governments of the European Higher Education Area (48 members including the 28 EU member states). The European Higher Education Area is the result of the political will of 48 countries which, during the last eighteen years, implement reforms on higher education on the basis of common key values—freedom of expression, autonomy for institutions, independent students unions, academic freedom, free movement of students and staff. Through this process, countries, institutions and stakeholders of the European area continuously adapt their higher education systems making them more compatible and strengthening their quality assurance mechanisms. The main goal is to increase staff and student mobility and to facilitate employability of university graduates. It proposes recommendations that are not legally binding. The first of these is a harmonised structure for all university degrees (including pharmacy) with a bachelor (3 years) followed by a master (2 years) degree. In this aspect, the Bologna agreement is in opposition to the EU directive. The latter requires a five-year, “tunnel” degree structure for pharmacy, i.e., a degree course that offers no possibility for intermediate entry or exit after accomplishment of a three-year bachelor period. 

The idea behind the Bologna recommendations is to improve student mobility, such as the development of tools to promote student exchange programmes like the European Credit Transfer and Accumulation System (ECTS). This provides credits to students for defined learning outcomes. ECTS are coupled to a Diploma Supplement that describes the nature, level, context, content and status of the studies that were successfully completed by a student. This system allows students to validate studies carried out at their host university by their home university. 

This paper looks at how the EU directive and the Bologna recommendations apply in a country, Austria that has been a member of the EU for quite some time (membership 1995). 

In order to place practice within the general health situation in Austria compared to Europe, it can be noted that life expectancy at birth (Table 1) in Austria is higher than the European average of 77 years. Healthy life expectancy (European average 68 years) is also higher. Furthermore, expenditure on health is slightly higher than the European average (9.4% of GDP). The fact all these health statistics are globally higher than the European average indicates that Austrian healthcare services such as pharmacy are of a high level.

## 2. Design

Information was obtained from academics and practicising pharmacists (the authors) and from internet sources (the university and association websites of the authors) on:

- pharmacy:
  o practice (community, hospital and industrial);
  o legislation;
  o education and training.
- harmonisation with the EU sectoral directive on pharmacy [2] and with the Bologna recommendations [6].

Electronic survey methodology was used; data was collected in 2010 and revised in 2017/18. We attempted at all times to collect objective (often numerical) data.

The information is presented in the form of tables in order to facilitate legibility. This type of presentation was developed in association with the journal’s editorial board and has been described in detail in a previous publication [7]. This format will ease the comparison of different EU countries by students and staff envisaging exchange programmes, and by researchers in pharmacy education and practice.

## 3. Evaluation and Assessment

### 3.1. Organisation of the Activities of Pharmacists, Professional Bodies

Table 2 provides details of the numbers and activities of community pharmacists and pharmacies in Austria. Items are expounded in the “comments” column.

Using the data in reference [1] and Table 2 it can be calculated that compared to the EU linear regression estimation (for definition and calculation see reference [1]), the ratio of the number of community pharmacists in Austria/population compared to the linear regression estimation = 0.90. Thus, the number of pharmacists per population is similar to the EU norm. The same comparison for community pharmacies produces a ratio of 0.54, lower than the EU norm. The number of pharmacists/pharmacy (4.3) is substantially higher than the EU average of 2.1 ± 0.7 (reference [1]).

The activities and occupations of pharmacists in Austria are similar to those of community pharmacists in other EU member states [1]. Certain specificities are seen, for example, concerning health care. In addition to expert advice, pharmacists offer health checks such as monitoring of blood pressure, blood sugar, cholesterol, weight and abdominal girth. Furthermore, regional or national campaigns with a focus on venous and lung function are regularly carried out. Every pharmacy can prepare magistral or officinal formulae preparations according to individual requirements, for example, for newly born and young children. Magistral or officinal formulae preparations are prescribed for skin diseases (e.g., ointments), where they make up 44% of such prescriptions. Many cough mixtures, eye drops and eye ointments are also prepared by pharmacists. Pharmaceutical preparation remains one of the core services of community pharmacies. Finally, pharmacies are involved in addictive drug substitution programmes. Every pharmacy is obliged to accept patients undergoing drug replacement therapy and this in the context of the rapid increase of the number of patients and the complex dispensing procedure.

Table 3 provides details of the numbers and activities of persons other than pharmacists working in pharmacies in Austria.

Turning to specialisation in pharmacy practice, Table 4 provides the numbers and activities of hospital pharmacists in Austria.

The number of pharmacists working in hospitals is lower than the EU average. The ratio of the actual number compared to the linear regression estimation is 0.67, (for definition and calculation see Reference [1]). The duties of hospital pharmacists are similar to those elsewhere in the EU [1].

There is no exact information on the numbers and activities of industrial pharmacists and pharmacists in other sectors in Austria. The total number of employees in the phrmaceutifcal industry is 14,140 (2017; [10]; the highest figure for the EU being Germany at 114,069). Regarding the pharmaceutical industry in Austria, there are 336 pharmaceutical and biotech companies with operations in Austria, of which 175 are R&D and manufacturers, and 161 are sales/service/suppliers [11]. The major multinational companies all have sales/distribution subsidiaries in Austria, and several also have R&D and/or manufacturing facilities. Austria has its own small and mid-size manufactures, including significant generics and homeopathic/natural remedy producers [12,13]. Austria has a positive pharmaceutical trade balance at +470 M€ [10], ranking 11th in the EU.

Table 5 provides information on professional associations for pharmacists in Austria.

### 3.2. Pharmacy Faculties, Students, and Courses

Table 6 provides details of pharmacy higher-education institutions (HEIs), staff and students in Austria.

A comparison to the EU average for staff shows that Austria has a low ratio 0.26 [1], although the number of pharmacy HEIs is similar to the EU norm at 0.97. Concerning teaching, it is interesting to note that Austrian pharmacy HEIs offer an introductory, orientation first term as do more and more EU pharmacy HEIs such as in France [14] and the UK [15].

Table 7 below contains details of specialisation electives.

It should be noted that a qualified person is defined by the EU pharmaceutical regulation medicinal products for human use that specify that no medicinal product can be released for sale or supply without certification by a qualified person that the product meets the relevant EU requirements. Qualified persons are often pharmacists in the EU.

For the profession of hospital pharmacist it is possible, but not mandatory to follow the *Weiterbildung zum Krankenhausfachapotheker* (training as a hospital pharmacist) 3-year course [16]. This is carried out at the work place and requires at least 240 h of additional study in areas such as management, team building, communication, production, and clinical pharmacy, etc. Each area is taught in 1–3 days seminars. For industrial pharmacists there are no undergraduate courses, only postgraduate courses.

Table 8 provides details of past and present changes in pharmacy education and training in Austria.

### 3.3. Subject Areas

Table 9 provides details of student units by subject area (for definitions of the subject areas see Reference [1]). 

It can be seen that the MEDISCI/CHEMSCI ratio in the master programme (year 4 and 5) is 2.83 thus reflecting the importance of medicinal science subjects. The overall ratio of MEDISCI/CHEMSCI (year 1–5) is 0.73. In the EU some HEIs such as Spain have a “balanced” course with a medicinal sciences/chemical sciences index of 1.2. Others have more “medical” courses such as Ireland and the Netherlands with indices of 2.6 and 1.6, respectively [1,17].

### 3.4. Impact of the Bologna Principles 

Table 10 provides details the various ways in which the Bologna declaration [3] impacts on the pharmacy HEIs of Austria.

Austria like other EU countries has adopted some elements of the Bologna system such as the use of ECTS and in the meantime also the 2 cycle system. The 4.5-year tunnel degree will remain in effect until 2021. 

### 3.5. Impact of European Union (EU) Directive 2013/55/EC 

Table 11 provides details the various ways in which the EC directive [2] impacts on pharmacy education and training in Austria.

Austria mainly conforms to the different aspects of the EU directive with a 4.5 year tunnel degree and a 1 year traineeship. 

## 4. Discussion and Conclusions

Pharmacy practice is harmonized with EU norms although Austria has fewer pharmacies than the EU average and more pharmacists per pharmacy. The latter may explain the substantial number of care elements provided by Austrian community pharmacies such as measurement of blood pressure and blood sugar, advice on components of healthy living, vaccinations and involvement in addictive drug substitution programmes. Furthermore in terms of life expectancy and expenditure on healthcare, Austria is at the top end of the EU scale. Another characteristic of Austrian community pharmacy practice is the fact that pharmacists are very involved in the production of magistral or officinal formulae preparations such as skin ointments.

Regarding specialisation, the Austrian hospital pharmacist is defined by both work place and specific duties that are different from those of a community pharmacist. There is however no undergraduate specialisation in hospital pharmacy; a 3-year training period in the form of continuous professional development assures specialisation. There are substantially fewer hospital pharmacies and pharmacists than the EU norm. There are master courses in industrial pharmacy but no specific job specification for industrial phrmacists. 

The Austrian education system is harmonised to that elsewhere in the EU. There are certain specificities however such as the existence of an orientation phase at the beginning of the phase course. There is a 1-year post-graduate traineeship which is twice as long as that laid down in the EU directive [2]. Thirdly whilst the number of pharmacy HEIs compared to the population is similar to the EU average, the number of staff is lower. Fourthly, the global degree course is oriented towards chemical subjects with a ratio of “medicinal subjects/chemical sciences” of 0.73. The 4th and 5th year master course is, however, highly oriented towards medicinal sciences (ratio = 2.83). The ratio in the EU is generally within the range of 0.7–1.7 [17]. This may be explained by the fact that pharmacy HEIs in Austria are part of the science faculty and as such are oriented towards pharmaceutical chemistry.

Concerning commitment to the the Bologna agreement (which was one of the main elements of the PHARMINE study), there is a two tier (bachelor and master) degree structure. Thus in the EU, some countries do have official bachelor and master courses (also France, Belgium) but generally only students with a bachelor in pharmacy are allowed to follow the master pharmacy course.

## Figures and Tables

**Table 1:** Health statistics for Austria [4,5].

Total Population	8,545,000
Life expectancy at birth (years)	82
Healthy life expectancy at birth (years)	72
Total health expenditure as % of GDP	10.8

**Table 2:** Numbers and activities of Austrian community pharmacists and pharmacies [8].

Item	Numbers	Comments
Community pharmacists	5822	31 December 2016 1468 persons/pharmacist
Community pharmacies	1352	31 December 2016 6320 persons/pharmacy. 4.3 pharmacists per pharmacy
Competences and roles of community pharmacists		Delivery of medicines o Advice on medicines, doctors’ prescriptions o Advice on the choice and use of non-prescription medicines o Check for adverse effects and interactions Preparation of magistral officinal formulae, production of medicines Health advice: giving up smoking, travel healthcare, diet, exercise, healthier lifestyle Health care: measurement of blood pressure, blood sugar, cholesterol, weight and abdominal girth, venous function, lung function Vaccination programmes: influenza, menigitis, hepatitis, pneumococci, meningococci, etc. Addictive drugs substitution programs Personal support for chronically ill persons Administrative tasks for health insurance funds: levying the prescription fee
Is ownership of a community pharmacy limited to pharmacists?	Yes	Only a pharmacist can own and manage a pharmacy. Partnerships are possible but the pharmacist must own at least 51%. Vertical integration is possible but restricted. No pharmacist is granted more than one license to manage a pharmacy. Multiple ownership of pharmacies, in the form of chains, is not allowed. A pharmacist may open a maximum of one branch pharmacy which operates under the supervision of the main pharmacy.
Rules governing the geographical distribution of pharmacies?	Yes	The establishment of a community pharmacy is regulated. Rules comprise geographic and demographic criteria. Establishment is also bound to a management permit, which means that the pharmacist must have at least five years of experience working in a pharmacy.
Are drugs and healthcare products available by other channels?	Partially	Around 890 doctors are allowed to dispense directly to their patients. Drugstores are allowed to sell a very restricted range of OTC medicines, e.g., herbal teas, remedies and cosmetics listed in the so-called “*Abgrenzungsverordnung*” (demarcation regulation). The mail order or online selling of non-prescription medicines–but not prescription-only medicines—is allowed within Austria.

**Table 3:** Numbers and activities of other personnel working in pharmacies in Austria [8,9].

Item	Number	Comments
Are persons other than pharmacists involved in community practice?	Yes	Pharmaceutical-commercial assistant Support personnel such as cleaning staff
Their titles and number(s)	5657 (2016)	Pharmaceutical-commercial assistants 0.97 assistants/pharmacist. 4.18 assistants/pharmacy.
Organisation providing and validating the E&T		Community pharmacy and vocational college
Duration of studies (years)		3 years after compulsory school 2 years for a high-school graduate
Subject areas		Basic modules in chemistry and in physics, healthcare, hygienic, management, economics, bookkeeping, etc.
Competences and roles		Pharma-commercial assistants do not have the right to dispense medicines. The main responsibilities of pharma-commercial assistants are: administration of products and assistance with preparation of pharmacy-produced medicines.

**Table 4:** Numbers and activities of hospital pharmacies and pharmacists.

Item	Number	Comments
Number of hospital pharmacists	412	There are 412 hospital pharmacists working in 45 hospital pharmacies in Austria. Only 15% of all hospitals operate their own hospital pharmacy [9].
Competences and roles of hospital pharmacists		The competences and roles of hospital pharmacists are defined by the *Law on how to operate a pharmacy*, which is an ordinance of the *Austrian Medicines Act* [8]: Provision and distribution of drugs, medical devices and other hospital-relevant goods (e.g., diagnostics, chemicals, reagents, etc.) Patient-specific compounding of individual prescriptions Checking of prescriptions for clarity and legal compliance Inspection of drug storage on wards Patient-oriented services–clinical pharmacy services with optimal, rational and safe pharmacotherapy Prescription in ambulatory care Pharma-economics Members of ethic committees Collaboration in clinical trials

**Table 5:** Professional associations for pharmacists in Austria.

Item		Comments
Registration of pharmacists	Yes	The Austrian Chamber of Pharmacists is responsible for the professional representation of all pharmacists, self-employed and salaried, who work in community and hospital pharmacies. Membership is compulsory. The Austrian Chamber of Pharmacists is designated by law to support and promote the professional, economic and social interests of pharmacists, to protect their professional honour, and to ensure that members comply with their professional duties (disciplinary jurisdiction).
Creation of community pharmacies and control of territorial distribution	Yes	The Regulation on the Operation of Pharmacies (*Apothekenbetriebsordnung* ABO [8]) states that pharmacies are to be checked by the local authority before they start operating and after that at least every five years. This control involves premises and equipment, as well as the products manufactured and stored in the pharmacy.
Ethical and other aspects of professional conduct	Yes	The Chamber of Pharmacists ensures the proper professional exercise and compliance with the ethical rules. Misconduct and breach of ethical rules may lead to disciplinary sanctions.
Involvement in HEI courses for pharmacists	Partial	The Chamber and its Regional Offices organise, finance and supervise the practical training of pharmacists. The Chamber of Pharmacists obliges pharmacists to undergo regular continuous professional development.

**Table 6:** Pharmacy higher education institutions (HEIs), staff, and students in Austria.

Item	Number/Reply	Comments
**HEIs in Austria**	3	
Public	3	Graz: https://pharmazie.uni-graz.at/en/research/pharmaceutical-technology-biopharmacy/ Vienna: http://merian.pch.univie.ac.at/pch/index.php Innsbruck: https://www.uibk.ac.at/pharmazie/phtech/
Attached to a science faculty	Yes	
Bachelor and master degrees?	Yes	Bachelor-Master system is implemented, with maintenance of the 4.5-year tunnel system until 2021.
**Austria**		
**Teaching staff**		
Number of teaching staff (nationals)	58	Graz: 18 Vienna: 26 Innsbruck: 14
International teaching staff		Honorary professorships were awarded to non-Austrian citizens—one honorary professor is a Swiss citizen. Visiting professors from several states have had time-limited teaching obligations.
**Entry requirements following secondary school**		
Specific pharmacy-related, national entrance examination	No	There are no national entrance examinations. However, there is a so called *study entrance and orientation phase* (first term), which students have to pass in order to continue their studies. Students have to pass three exams (16 ECTS) on the following: Introductory lecture series on the main subjects of the pharmacy studies Biology for pharmacists General chemistry for pharmacists
Is there a national *numerus clausus*?	No	
**Advanced entry**		
At which level?		Pharmacy students after examination of equivalency of their studies. Doctoral students from pharmacy and pharmacy related disciplines after examination of equivalency of their degree.
**Fees per year**		
EU students	0	
Non EU students	380 €	

**Table 7:** Specialisation electives in pharmacy HEIs.

Item	Response	Comments
Do HEIs provide specialized courses?	Partial	There are is one diploma graduate program and several doctorate graduate programs with specific profiles. Graz: Master Programme in Chemical and Pharmaceutical Engineering (https://pharmazie.uni-graz.at/en/study-teach/cpe-master-program/). Vienna: A training course for qualified persons. Innsbruck: master courses in natural sciences, agriculture, environment, etc. (https://www.mastersportal.eu/universities/4892/faculty-of-chemistry-and-pharmacy.html#StudyListing).
In which years?	5th year on	Courses are postgraduate.

**Table 8:** Past and present changes in education and training in Austrian pharmacy HEIs.

Item		Comments
Have there been any major changes since 1999?	Yes	There is regular adaptation of the study programme organised at a national level, prepared mainly by the *Curriculare Arbeitsgruppen* in co-operation with the Austrian Pharmaceutical Society and with involvement of the Chamber of Pharmacists.
Are any major changes envisaged before 2019?	Yes	There will be regular adaptation of the study programme organised at a national level prepared mainly by the *Curriculare Arbeitsgruppen* in co-operation with the Austrian Pharmaceutical Society and with involvement of the Chamber of Pharmacists. The adaptation of pharmacy curricula according to the Bologna criteria was an issue of discussion in Austria and since 2015 the bachelor-master system was adapted by all (three) Austrian universities. The 4.5-year tunnel degree will remain in effect until 2021.

**Table 9:** Student units by subject area (ECTS).

Subject Area	Year 1	Year 2	Year 3	Year 4	Year 4	Total
CHEMSCI	29	27	22	7	5	90
PHYSMATH	7	0	0	0	0	7
BIOLSCI	7	17	12	19	0	55
PHARMTECH	1	0	20	20	0	41
MEDISCI	16	16	0	14	20	66
LAWSOC	0	0	0	0	2	2
GENERIC	0	0	6	0	8 + 25 *	39
Total	60	60	60	60	60	300
%	20	20	20	20	20	100

CHEMSOC: chemical sciences; PHYSMATH: physical and mathematical sciences; BIOLSCI: biological sciences; PHARMTECH: pharmaceutical technology; MEDISCI: medicinal sciences; LAWSOC: law and social sciences; GENERIC: generic competences. *: Master thesis.

**Table 10:** Ways in which the Bologna declaration impacts on Austrian pharmacy HEIs.

Item		Comments
“Comparable degrees with diploma supplement”	Yes	The Austrian universities consider the “magister” as equivalent education to a Master Degree according to Bologna.
“Two main cycles (B and M) with entry and exit at B level”	No	
“European Credit Transfer System (ECTS) system of credits”	Yes	Lectures are ECTS weighted.
“Addressing obstacles to mobility”		No obstacles. Traditional involvement in Erasmus programmes.

**Table 11:** Ways in which elements of the European Commission (EC) directive (left column) impact on Austrian pharmacy HEIs.

Item	Comments
“Evidence of formal qualifications as a pharmacist shall attest to training of at least five years’ duration…”	This is implemented.
“…four years of full-time theoretical and practical training at a university or at a higher institute of a level recognised as equivalent, or under the supervision of a university;”	This is implemented.
“…six-month traineeship in a pharmacy which is open to the public or in a hospital, under the supervision of that hospital’s pharmaceutical department.”	This is a 1-year period. The post-university training programme should rather be more structured than be shortened.

## References

[1] Atkinson J., Rombaut B. (2011). The 2011 PHARMINE report on pharmacy and pharmacy education in the European Union. Pharm. Pract..

[2] The European Commission Directive 2013/55/EU on Education and Training for Sectoral Practice Such as That of Pharmacy. http://eur-lex.europa.eu/legal-content/FR/TXT/?uri=celex:32013L0055.

[3] The European Higher Education Area (EHEA)—Bologna Agreement of Harmonisation of European University Degree Courses. http://www.ehea.info/.

[4] World Health Organisation (WHO) Global Health Observatory Statistics 2016. http://www.who.int/gho/publications/world_health_statistics/2016/Annex_B/en/.

[5] European Health Information Gateway Total Health Expenditure as % of GDP. https://gateway.euro.who.int/en/indicators/hfa_566-6711-total-health-expenditure-as-of-gdp/.

[6] Erasmus Programme for Student and Staff Exchange in the EU. https://info.erasmusplus.fr/.

[7] Atkinson J. (2017). The Country Profiles of the PHARMINE Survey of European Higher Educational Institutions Delivering Pharmacy Education and Training. Pharmacy.

[8] (2016). Verordnung der Bundesministerin für Gesundheit und Frauen über den Betrieb von Apotheken und Ärztlichen und Tierärztlichen Hausapotheken (Apothekenbetriebsordnung 2005—ABO 2005).

[9] Die Österreichische Apotheke in Zahlen. The Austrian Pharmacy—Facts and Figures. apps.who.int/medicinedocs/documents/s17236de/s17236de.pdf.

[10] EFPIA—The European Federation of Pharmaceutical Industries and Associations: The Pharmaceutical Industry in Figures, Key Data 2017. https://www.efpia.eu/publications/downloads/.

[11] Austria—Drugs and Pharmaceuticals Export.gov USA. https://www.export.gov/article?id=Austria-Drugs-and-Pharmaceuticals.

[12] Austrian Life Sciences Directory. http://www.lifesciencesdirectory.at/.

[13] Pharmig, Association of the Austrian Pharmaceutical Industry. http://www.pharmig.at/DE/Publikationen/Daten%20%20Fakten/2017/Daten++Fakten+2017.aspx.

[14] (2017). 1re Année Commune Aux Etudes de Santé.

[15] MPharm Pharmacy with a Foundation Year Manchester University, UK. https://www.bmh.manchester.ac.uk/study/undergraduate/courses/2018/mpharm-pharmacy-with-a-foundation-year/?pg=2#course-profile.

[16] (2018). Arbeitsgemeinschaft Österreichischer Krankenhausapotheker.

[17] Atkinson J., De Paepe K., Sánchez Pozo A., Rekkas D., Volmer D., Hirvonen J., Bozic B., Skowron A., Mircioiu C., Marcincal A. (2015). Does the Subject Content of the Pharmacy Degree Course Influence the Community Pharmacist’s Views on Competencies for Practice?. Pharmacy.

